# New Books

**Published:** 2007-03

Acid in the Environment

Gerald R. Visgilio, Diana M. Whitelaw, eds.

New York:Springer, 2007. 332 pp. ISBN: 978-0-387-37561-8, $89.95

Apoptosis, Cell Signaling, and Human Disease

Rakesh Srivastava

Totowa, NJ:Humana Press, 2006. 384 pp. ISBN: 1-58829-677-6, $145

Arsenic in Soil and Groundwater

P. Bhattacharya, A. Mukherjee, R. Zevenhoven, J. Bundschuh, R. Loeppert, eds.

Burlington, MA:Elsevier, 2007. 496 pp. ISBN: 0-444-51820-7, $120

Biology of the Nitrogen Cycle

H. Bothe, S. Ferguson, W.E. Newton, eds.

Burlington, MA:Elsevier, 2007. 452 pp. ISBN: 0-444-52857-1, $120

Cancer Genomics and Proteomics

Paul B. Fisher

Hackensack, NJ:World Scientific Publishing Co., 2007. 481 pp. ISBN: 1-58829-504-4, $99.50

Depleted Uranium: Properties, Uses, and Health Consequences

Alexandra C. Miller

Boca Raton, FL:CRC Press, 2006. 288 pp. ISBN: 0849330475, $149.95

Environmental Modeling: A Practical Introduction

Mike Barnsley

Boca Raton, FL:CRC Press, 2007. 432 pp. ISBN: 0-4153-0054-1, $99.95

Environmental Policy and Public Health

Barry L. Johnson

Boca Raton, FL: CRC Press, 2006. 496 pp. ISBN: 0-8493-8434-6, $79.95

Environmental Value Transfer: Issues and Methods

Stále Navrud, Richard Ready, eds.

New York:Springer, 2007. 290 pp. ISBN: 978-1-4020-4081-8, $149

Fundamentals of Data Mining in Genomics and Proteomics

Werner Dubitzky, Martin Granzow, Daniel P. Berrar, eds.

New York:Springer, 2007. 282 pp. ISBN: 978-0-387-47508-0, $79.95

Genomic Approaches for Cross-Species Extrapolation in Toxicology

William H. Benson, Richard T. Di Giulio

Boca Raton, FL:CRC Press, 2006. 216 pp. ISBN: 1-4200-4334-X, $99.95

Handbook of Globalization and the Environment

K.V. Thai, D. Rahm, J.D. Coggburn

Boca Raton, FL:CRC Press, 2007. 616 pp. ISBN: 1-5744-4553-7, $99.95

Particle Toxicology

Ken Donaldson, Paul Borm

Boca Raton, FL:CRC Press, 2006. 434 pp. ISBN: 0-8493-5092-1, $159.95

Renewable Resources and Renewable Energy: A Global Challenge

Mauro Graziani, Paolo Fornasiero

Boca Raton, FL:CRC Press, 2006. 384 pp. ISBN: 0-8493-9689-1, $129.95

Thermodynamics, Solubility and Environmental Issues

Trevor Letcher, ed.

Burlington, MA:Elsevier, 2007. 454 pp. ISBN: 0-444-52707-9, $120

Water and Development in China: The Political Economy of Shanghai Water Policy

Seungho Lee

Hackensack, NJ:World Scientific Publishing Co., 2006. 332 pp. ISBN: 981-256-819-0, $68

Who Owns the Water?

K. Lanz, L. Müller, C. Rentsch, R. Schwarzenbach, eds.

New York:Springer, 2007. 535 pp. ISBN: 978-3-03778-018-3, $60

